# Signal Disruption Leads to Changes in Bacterial Community Population

**DOI:** 10.3389/fmicb.2019.00611

**Published:** 2019-03-29

**Authors:** Michael Schwab, Celine Bergonzi, Jonathan Sakkos, Christopher Staley, Qian Zhang, Michael J. Sadowsky, Alptekin Aksan, Mikael Elias

**Affiliations:** ^1^Department of Biochemistry, Molecular Biology and Biophysics, University of Minnesota, Twin Cities, St. Paul, MN, United States; ^2^Biotechnology Institute, University of Minnesota, Twin Cities, St. Paul, MN, United States; ^3^Department of Mechanical Engineering, University of Minnesota, Twin Cities, St. Paul, MN, United States; ^4^Department of Surgery, University of Minnesota, Twin Cities, St. Paul, MN, United States; ^5^Department of Soil, Water, and Climate, University of Minnesota, Twin Cities, St. Paul, MN, United States; ^6^Department of Plant and Microbial Biology, University of Minnesota, Twin Cities, St. Paul, MN, United States

**Keywords:** quorum sensing, lactonase, biofilm, microbial community, silica encapsulation

## Abstract

The disruption of bacterial signaling (quorum quenching) has been proven to be an innovative approach to influence the behavior of bacteria. In particular, lactonase enzymes that are capable of hydrolyzing the *N-*acyl homoserine lactone (AHL) molecules used by numerous bacteria, were reported to inhibit biofilm formation, including those of freshwater microbial communities. However, insights and tools are currently lacking to characterize, understand and explain the effects of signal disruption on complex microbial communities. Here, we produced silica capsules containing an engineered lactonase that exhibits quorum quenching activity. Capsules were used to design a filtration cartridge to selectively degrade AHLs from a recirculating bioreactor. The growth of a complex microbial community in the bioreactor, in the presence or absence of lactonase, was monitored over a 3-week period. Dynamic population analysis revealed that signal disruption using a quorum quenching lactonase can effectively reduce biofilm formation in the recirculating bioreactor system and that biofilm inhibition is concomitant to drastic changes in the composition, diversity and abundance of soil bacterial communities within these biofilms. Effects of the quorum quenching lactonase on the suspension community also affected the microbial composition, suggesting that effects of signal disruption are not limited to biofilm populations. This unexpected finding is evidence for the importance of signaling in the competition between bacteria within communities. This study provides foundational tools and data for the investigation of the importance of AHL-based signaling in the context of complex microbial communities.

## Introduction

Bacterial quorum sensing (QS) is among the most prominent and studied communication systems used by bacteria (Bassler, 1999). Numerous bacteria produce and utilize chemical signal molecules to coordinate their behavior in a cell density-dependent manner (Miller and Bassler, 2001; LaSarre and Federle, 2013). Bacterial QS has been shown to also regulate virulence and biofilm formation (LaSarre and Federle, 2013). Biofilms are comprised of a hydrated matrix of polysaccharides, proteins and nucleic acids that ultimately allow bacteria to attach to surfaces and live in complex community structures (Costerton et al., 1999). These structured communities enable a multicellular-like existence that is distinct from the planktonic state (Stewart and Costerton, 2001).

Quorum quenching (QQ) relates to all processes that can interfere with QS (McClean et al., 1997). QQ is a strategy that is not aimed at killing bacteria or at limiting growth, but rather at controlling or changing the expression of different functions (Uroz et al., 2009). Consequently, QQ enzymes are naturally capable of interfering with this QS *via* the enzymatic degradation of autoinducer molecules (Zhang, 2003; LaSarre and Federle, 2013). This has been studied in the case of autoinducer-1, *N*-acyl homoserine lactones (AHLs) (Dong et al., 2000, 2001; Oh et al., 2012; Kim et al., 2013; Bzdrenga et al., 2016). Indeed, the disruption of bacterial signaling using QQ enzymes was previously shown to inhibit the production of virulence factors and biofilm production by numerous pathogens, both *in vitro* (Dong et al., 2000; Chow et al., 2014; Hraiech et al., 2014; Guendouze et al., 2017; Zhang et al., 2017) and *in vivo* (Dong et al., 2000; Hraiech et al., 2014). These properties have been instrumental in making QQ enzymes prime candidates for bacterial control in numerous fields of application. However, to achieve this goal, effort is required to overcome practical issues with use of QQ enzyme technology, such as low activity levels, activity at low or high temperatures, environmental stability, and production costs (Lee et al., 2016; Rémy et al., 2016).

A promising candidate to overcome the intrinsic limitations in current enzymes is the lactonase *Sso*Pox, isolated from the hyperthermophilic crenarcheon *Sulfolobus solfataricus* (Merone et al., 2005; Elias et al., 2007, 2008). This enzyme belongs to the Phosphotriesterase-Like Lactonase family (Afriat et al., 2006; Elias and Tawfik, 2012), and naturally hydrolyzes a broad range of AHLs, from C6 AHL to 3-oxo C12 AHL (Hiblot et al., 2013). The *Sso*Pox was shown to disrupt bacterial QS *in vitro* and *in vivo* (Hraiech et al., 2014; Guendouze et al., 2017). Additionally, this lactonase was reported to be catalytically active over a wide range of temperatures, from -19°C to 70°C (Merone et al., 2005; Rémy et al., 2016). Interestingly, this lactonase exhibits exceptional thermal stability (*T*_m_ = 106°C), resistance to denaturing agents, organic solvents, detergents, radiation, and proteases (Hiblot et al., 2012a; Rémy et al., 2016). A crystal structure of *Sso*Pox revealed the critical importance of residue W263, interacting with the bound lactone ring of the AHL molecule (Elias et al., 2007, 2008; Del Vecchio et al., 2009). Mutation of W263I allowed for generation of variants with even greater lactonase catalytic activity (Hiblot et al., 2013; Jacquet et al., 2017).

While the substrate specificity of several lactonases has been determined (Hiblot et al., 2012b, 2013, 2015; Bzdrenga et al., 2014; Mascarenhas et al., 2015; Tang et al., 2015; Bergonzi et al., 2016, 2017), the types of bacteria that can be controlled by these enzymes is unclear. Indeed, AHL-based QS and effects of QS interference were mostly described in Gram-negative bacteria (Chow et al., 2014; Hraiech et al., 2014; Ivanova et al., 2015a,b; Guendouze et al., 2017). Other studies report activity of lactonase of bacterial strains that are not known as using AHLs (Ivanova et al., 2015a; Mayer et al., 2015). The presence of bacteria expressing lactonases was shown to reduce biofouling in a membrane bioreactor (MBR) (Oh et al., 2012; Kim et al., 2013, 2015), as well as affect the community structure of microbes attached to the membrane (Jo et al., 2016). Despite this, tools and insights are missing to adequately decipher the mechanisms underlying these observations.

In order to determine the effects of AHL degradation in the context of a complex microbial community, we used a silica gel, bead-based, bioencapsulation technique. Silica is a cytocompatible material in which bacteria or their enzymes can be physically confined, retained within the matrix, and protected from the environment (Reátegui et al., 2012; Mutlu et al., 2013, 2015; Aukema et al., 2014; Sakkos et al., 2016, 2017). Here, we used encapsulated *Escherichia coli* cells overexpressing the lactonase *Sso*Pox W263I to produce enzymatically active beads. Because SsoPox is a hydrolase, and do not need any other recycling co-factor than water molecules, it does not need the cells to remain alive to maintain its catalytic activity. In fact, in this strategy, cells are used as “bags of enzymes.” Encapsulation of bacteria overexpressing stable, engineered lactonases combines the intrinsic properties of the *Sso*Pox enzyme, the lower production costs associated with the use of cells instead of purified enzyme, and a robust, permeable silica structure facilitating the integration of this enzyme in water treatment systems.

Catalytically active capsules were used as an enzymatic filtration matrix to degrade AHL signaling molecules produced by a complex soil microbial community cultured in a recirculating system. We determined that the presence of the lactonase in the filtration beads leads to a very significant reduction of biofilm formation over the course of the experiment (21 days) and that this reduction is associated with a change of the microbial population forming the biofilm. This experimental system opens up a new way to study the importance of bacterial signaling in complex microbial communities, the effects of signal disruption using lactonases, and highlights the potential of these enzymes to serve in water treatment processes, including a recirculating system.

## Materials and Methods

### Preparation of Silica Lactonase-Containing Beads

The QQ lactonase *Sso*Pox W263I and a negative control protein, an inactive mutant SsoPox 5A8 [carrying the mutations V27G/P67Q/L72C/Y97S/Y99A/T177D/R223L/L226Q/L228M/W263H, obtained previously (Hiblot et al., 2013; Bergonzi et al., 2018)] were overexpressed in *E. coli* BL21-pGro7 as previously described using the autoinducing media ZYP (Hiblot et al., 2012a, 2013; Jacquet et al., 2017). The use of the 5A8 mutant allows for the control of the used plasmids and protein expression, allowing us to link observations to the enzymatic activity of SsoPox W263I. Cultures were grown to OD_600__nm_ = 0.8 at 37°C, while shaking at 200 RPM, and after overnight induction of the lactonase (18°C, 0.2% L-arabinose, 200 RPM shaking), cells overexpressing proteins were centrifuged at 4,400 ×*g* for 20 min at 4°C. Cells were re-suspended in 100 mM potassium phosphate buffer, pH 7, at a concentration of 0.4 g/mL wet weight to provide 0.2 g/mL for the 1× lactonase beads. Gel beads (1 mm diameter) containing the lactonase/control bacteria cultures were made using a dripping method while gelation occurred, using a method similar to a previously used protocol (Mutlu et al., 2013). Polyethylene glycol (PEG, 400 mg) with an average molecular weight of 10,000 Da, was mixed with 4 mL acetic acid (0.01 M) until the PEG dissolved. A 2.5 mL aliquot of tetramethyl orthosilicate (TMOS) was added and allowed to stir for 30 min until the solution became clear. One milliliter of cell suspension (0.2 g/mL) was mixed with the PEG/TMOS/acetic acid solution and gelation occurred within a few minutes. The bacteria-encapsulated beads (8 mL) were added directly to empty 10 mL chromatography columns to create filtration cartridges. Encapsulated bacteria can remain variable for several weeks. A report on encapsulation using similar gels show a reduction of ∼93% of viable *E. coli* cells after 3 weeks (Reátegui et al., 2012). Cell leakage from the gel is possible, but was previously found to be non-significant in similar gels (Reátegui et al., 2012). A filter (GE Healthcare) at the outlet of the column ensured the amount of beads present in the column would be constant throughout the duration of the experiment. Two different types of bacteria-encapsulated silica beads were produced, (1) beads where *E. coli* cells overexpressing the lactonase *Sso*Pox W263I were entrapped (*lactonase beads)* and (2) beads where *E. coli* cells overexpressing the control protein (inactive mutant 5A8) were entrapped (*control beads*). These beads were used to produce three distinct filtration cartridges: (a) the 2× lactonase cartridge, containing only 8 mL of *lactonase beads*, (b) a control cartridge containing only 8 mL of *control beads,* and (c) a 1× lactonase cartridge containing a 1:1 ratio of *lactonase beads* and *control beads* (4 mL of each).

### Kinetic Assays of Lactonase in Silica Gel

Lactonase-containing silica gel solution, and the control protein, were poured into individual wells of 96 well microplates and allowed to solidify. Enzyme activity was quantified over a ∼7 months period (28 weeks) in buffer. Each well contained 75 μL of gel and was stored at 4°C in the presence of the *pte buffer* (50 mM HEPES, pH 8, 150 mM NaCl, 0.2 mM CoCl_2_) or the *lactonase buffer* (2.5 mM Bicine pH 8.3, 150 mM NaCl, 0.2 mM CoCl2, 0.2 mM cresol purple, and 0.5% DMSO). The gel plus the buffer volume was 200 μL providing a 6.2 mm path length. Enzyme kinetics were measured by using a microplate reader (Synergy HT, BioTek, United States; Gen5.1 software), facilitated by a high level of gel transparency. The kinetics of lactonase activity were determined as previously described (Hiblot et al., 2012b, 2015; Bergonzi et al., 2016, 2017). Lactonase activity was expressed in enzymatic units defined as μM of substrate hydrolyzed per min per mg of cells (wet weight). All kinetic measurements were performed as triplicates. The activities of lactonase and phosphotriesterase were corrected by subtracting activities control gels (containing *E. coli* cells overexpressing mutant 5A8). The chromogenic substrate paraoxon was used as a proxy for the enzyme activity in order to evaluate the durability of gels over time. Assays were done as previously described (Hiblot et al., 2012a; Gotthard et al., 2013; Jacquet et al., 2017) and were performed using 10 μL of 20 mM paraoxon (1 mM final concentration) and a 200 μL final reaction volume. The paraoxon degradation product (paranitrophenolate) was directly measured at 405 nm (𝜀 = 17,000 M^-1^ cm^-1^ at pH 8.0). Activity over time was normalized to the measured activity at day 0.

### Flow-Through Recirculating Bioreactor System

**FIGURE 1:**
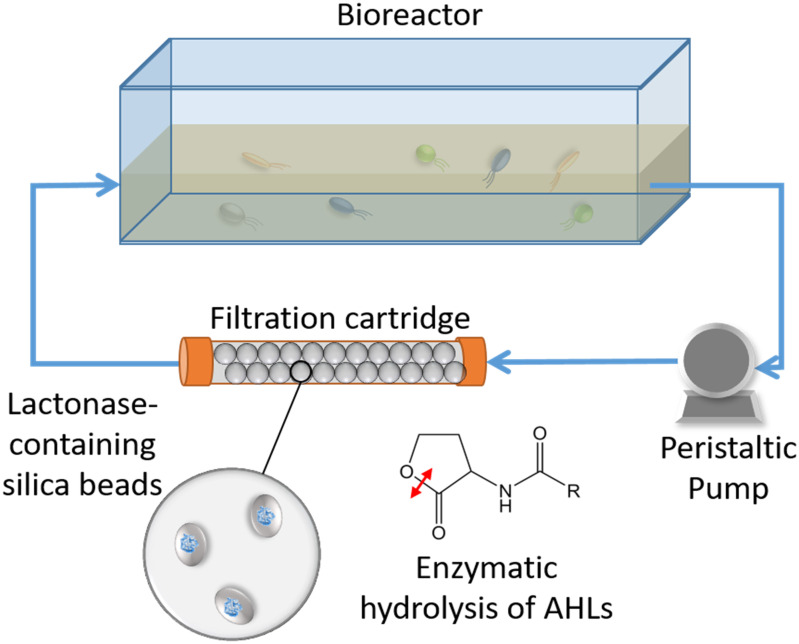
Schematic representation of the experimental system. Bacterial communities are cultured in a tank vessel. A peristaltic pump is pumping the culture media through a filtration cartridge made of silica beads. The beads are entrapping *E. coli* cells that overproduced an engineered quorum quenching lactonase. As the system operates, the *N*-acyl homoserine lactone molecules (AHLs) produced by cultured bacteria are enzymatically degraded by the filtration cartridge.

The flow-through system used in this study consisted of three 3 L tanks set up in parallel. The parallel circuit was achieved through the use of a multi-channel peristaltic pump (Masterflex L/S, Cole-Parmer, United States) (Figure 1 and Supplementary Figure S1). A peristaltic pump provided an even flow rate of 18 mL/min to each tank. The flow-through filtration cassette consists of a 10 mL chromatography column filled with QQ gel beads or control beads. Each tank contained three liters of 15× diluted LB medium in water. A pre-separated 96-stripwell plate was submerged to the bottom so that individual wells could be harvested for biofilm quantification or DNA extraction. At the bottom of each bioreactor were 22 mm square microscope cover slips to be later used for biofilm imaging. For the inoculum, ∼5 g of soil (disturbed Waukegan Silt loam; sampled in march) was suspended in 40 mL of water. Soil community was chosen as a model system to this study. The suspension was centrifuged for 5 min at 500 ×*g* and 200 μL of the cloudy supernatant was added to 30 mL of LB medium and allowed to grow for 16 h at 37°C, with shaking at 200 RPM. A 10 mL aliquot of this culture was inoculated into each tank and the system was allowed to run for 21 days at room temperature. During this time, measurements were taken every day to monitor OD_600__nm_ and pH of the water, and amount of biofilm present on multiple coverslip surfaces.

A second, independent experiment was performed similarly using soil samples from the same location (sampled in October) using duplicate bioreactors. The system was allowed to run for 10 days, and samples were taken at days 3 and 10.

Attempts to measure the AHLs concentration in the culture media using the sensor *Chromobacterium violaceum* CV026 have failed, due to the apparent toxicity of the supernatants to the biosensor.

### Effects of the Lactonase on a Complex Planktonic Community

The soil inoculum was used to inoculate 5 mL triplicate cultures (15× diluted LB medium) in 50 mL conical tubes. Tubes were incubated at 25°C and treated by adding to the culture media either inactive mutant SsoPox 5A8 enzyme or the improved mutant SsoPox W263I enzymes to a final concentration of 0.5 mg/mL. Samples were collected for DNA extraction after 3 and 7 days. In this experimental conditions, no biofilm could be visualized or quantified using crystal violet.

### Biofilm Quantification

The submerged 96-stripwell plates were pre-broken so that individual wells could be extracted every day for measurements. Individual wells were extracted, in duplicate, for biofilm formation by using crystal violet biofilm quantification at 550 nm as previously described (Hraiech et al., 2014). To assess planktonic growth in the tanks, optical density of 200 μL samples were measured by using a 96 well plate spectrophotometer at 600 nm.

### pH Measurements

pH values were measured with a meter (Orion Star A214, Thermo Fisher Scientific, United States). The pH of each bioreactor was monitored throughout the experiment (Supplementary Figure S2).

### Sample Preparation for Imaging

Microscope cover glass samples were harvested for biofilm visualization analysis by using a Zeiss confocal microscope (West Germany, Cell Observer SD). The cover slips were fixed with 2% paraformaldehyde in 1× PBS for 1 h at room temperature, rinsed twice with 1× PBS, and fixed in a solution of 50% 1× PBS containing 50% EtOH. Samples were stored at -20°C for later processing. For imaging analyses, the stored samples were washed twice with 1× PBS and stained with 1× SYBR Gold nucleic acid stain (Thermo Fisher, United States) for 10 min, washed with 100% EtOH, and mounted onto microscope slides for fluorescence analysis. A 1:4 mixture of Citifluor:Vectashield was used as the mounting medium.

### Microbial Community Analysis

Submerged wells from the stripwell plates were drained of excess cells/water and biofilm was scraped from the polypropylene well and into Powerbead^®^ tubes for DNA extraction (DNeasy PowerSoil^®^ DNA Extraction Kit, QIAGEN, Hilden, Germany). Purified DNA samples were submitted to the University of Minnesota’s Genomics Center for 16S rRNA sequencing on the MiSeq platform at the University of Minnesota Genomics Center (Minneapolis, MN, United States). Each sample underwent amplification, dual-indexing, normalization, pooling, size selection, and a final QC prior to sequencing. The V4 region of the 16S rRNA gene was amplified by using primer set 515f (5′ – GTG CCA GCM GCC GCG GTA A – 3′) and 806R (5′ – GGA CTA CHV GGG TWT CTA AT – 3′). Negative (sterile water) controls were included throughout amplification and sequencing. Sequencing data were deposited to the Sequence Read Archive (SRA) under the accession number SRP156219.

### Sequencing Data Analysis

All samples were processed using Mothur v1.35.1 (Schloss et al., 2009). Forward and reverse reads were paired-end joined using fastq-join software (Aronesty, 2013), and subjected to quality trimming using parameters previously described (Staley et al., 2015). High quality sequences were aligned against the SILVA database v.119.v4 (Pruesse et al., 2007), subjected to a 2% pre-cluster to remove sequence errors (Huse et al., 2010), and chimeras were removed using UCHIME v. 4.2.40 (Edgar et al., 2011). Operational taxonomic units (OTUs) were classified at 97% similarity using the furthest-neighbor algorithm and classified against the Ribosomal Database Project v.11.5 (Cole et al., 2008). For each sample, 1,250 sequences were used for final analyses. Alpha diversity indices were analyzed through the Mothur v1.35.1 program. Genus-level identification was achieved for the composition of the bacterial community. Analysis of similarity (ANOSIM) and analysis of molecular variances (AMOVA) were used to evaluate the beta diversity (community composition) among samples using Bray–Curtis dissimilarity matrices (BC) (Bray and Curtis, 1957; Excoffier et al., 1992; Clarke, 1993). Ordination of Bray–Curtis matrices was performed using principal coordinate analysis (PCoA) to further analyze diversity of sample days throughout the tank (Anderson and Willis, 2003). Pearson correlation was performed using Mothur v1.35.1 program to evaluate the correlation between the genera abundance under temporal change in different treatments. To visualize the distribution of taxonomies and diversities in microbial communities among the samples, “ggplot2” package in R v3.3.1 was used with rarefied relative abundance and OTUs at genus level (Aislabie et al., 2013). All of statistical analyses were done using α = 0.05. Graphpad prism software was used to calculate student unpaired, two-tailed *t*-test values.

## Results and Discussion

### Engineered Lactonase-Expressing Cells Entrapped *in silica* Capsules

Silica encapsulation is a method of choice for entrapping enzymes or cells due to their compatibility with biological molecules, mechanical properties, durability, stability, cost, and easy synthesis. Silica gels have been previously used to encapsulate bioreactive bacteria for bioremediation (Reátegui et al., 2012; Aukema et al., 2014; Sakkos et al., 2016, 2017). While most encapsulated bacteria may remain viable through the process of making the gels (Benson et al., 2018), it is likely to be unnecessary in this study, since the lactonase *Sso*Pox is a metalloenzyme that only requires a water molecule as the nucleophile for the hydrolytic reaction (Elias et al., 2008). Therefore, cells can be viewed as “bags of enzymes” that disrupt the AHL signaling molecules produced by bacteria.

**FIGURE 2:**
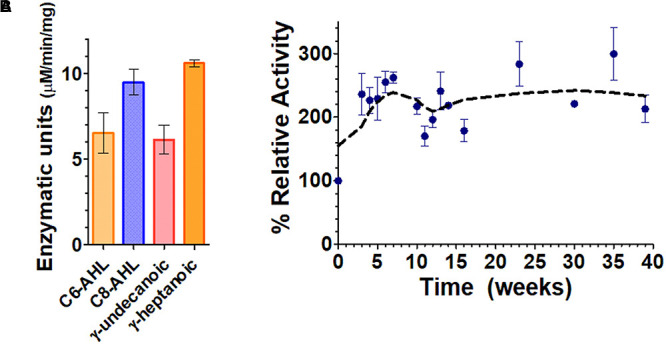
Functionalized silica gel enzymatic activities and durability. **(A)** Lactone hydrolysis activity of the engineered silica gels containing the lactonase *Sso*Pox W263I using lactones with short aliphatic chains, i.e., γ-heptanoic lactone (γ-heptanolactone) and C6-AHL, as well as with lactones with longer aliphatic chains, i.e., C8-AHL and γ-undecanoic lactone as substrates. Lactonase activity is expressed in enzymatic units defined as μM of substrate hydrolyzed per min per mg of cells. **(B)** Activity of the enzymatic silica gels over time, using the chromogenic substrate paraoxon as a proxy for enzyme activity.

The engineered silica gels showed catalytic activity against lactones including lactones with short aliphatic chains (i.e., C6-AHL and γ-heptanoic lactone) and lactones with long acyl chains (i.e., C8-AHL and γ-undecanoic lactone). This observation is consistent with the enzyme activity in solution (Figure 2A), that is reported to degraded AHLs ranging from C6 to 3-oxo C12 AHL, and oxonolactone of numerous chain lengths with similar catalytic efficiencies (Hiblot et al., 2013). The measured activity demonstrates that the lactonase overproduced in *E. coli* cells is active inside the beads, and that different types of lactones can access its active site. The lactonase assay used in this study was pH-based and was previously described by us (Hiblot et al., 2012b; Bergonzi et al., 2016, 2017). While this assay allows for the monitoring of the lactone ring opening (generating a proton), it requires significant optimization of the activity buffer for each measurement due to the buffering capacity of the gel.

In order to better and more conveniently evaluate the durability of the silica gels over time, we used the chromogenic substrate paraoxon as a proxy for *Sso*Pox activity, as previously reported (Elias et al., 2008; Hiblot et al., 2012a, 2013).

Results in Figure 2B show that the lactonase-containing gel remains active for at least 39 weeks (∼9 months) in solution. This is 5 months longer than previous studies on atrazine degradation that were performed with a different enzyme but in similar conditions (Reátegui et al., 2012). The observed durability is consistent with the extreme stability of *Sso*Pox W263I, that remains stable for >300 days (∼10 months) at 25°C as a purified protein sample (Rémy et al., 2016). Interestingly, the activity of the enzymatic gel at T_0_ increases over the course of the first 5 weeks of the experiment (approximately threefold). This may be caused by a change in the structure or porosity of the silica gel that could lead to an increased diffusion of the substrate into the enzyme active sites and may suggest that our current gel formulation could be optimized in future studies. Our success in obtaining silica gels containing engineered, overexpressed lactonases opens up a lot of new possibilities to study signal disruption in microbial communities.

Control of expression level, and the ability to engineer the lactonase, will be useful to optimize QQ in complex contexts. Additionally, because this technology in practice does not require purified enzyme, it may allow for the production of highly potent, specific beads to inhibit biofilms and biofouling in water filtration systems, at a low cost.

### Silica Beads Containing Lactonase Enzyme Inhibit Biofilm Formation of Complex Microbial Communities in a Water Recirculating System

In this study, a water recirculating system was used to examine the influence of lactonase on soil bacterial community structure in planktonic cells. The water within the tank was pumped through a filtration cartridge (Figure 1 and Supplementary Figure S1) containing different porous, lactonase-containing silica capsules (see section “Materials and Methods”). This experimental design was based on the hypothesis that water soluble AHLs produced by the microbial community growing in the tank would be filtered through the cartridge, and degraded by the lactonase enzyme.

The effects of the lactonase enzyme in the filtration cartridge on the microbial community was monitored at different levels by examining the pH of the tank medium and the optical density at 600 nm. The pH of the tank media increased from a starting value of ∼6.2 to a final value of ∼8.0 in all three experimental setups (Supplementary Figure S2A). Similarly, the OD_600__nm_, used as a proxy for cell density, slightly increased over the course of the experiment in a similar fashion in the three bioreactors (Supplementary Figure S2B).

**FIGURE 3:**
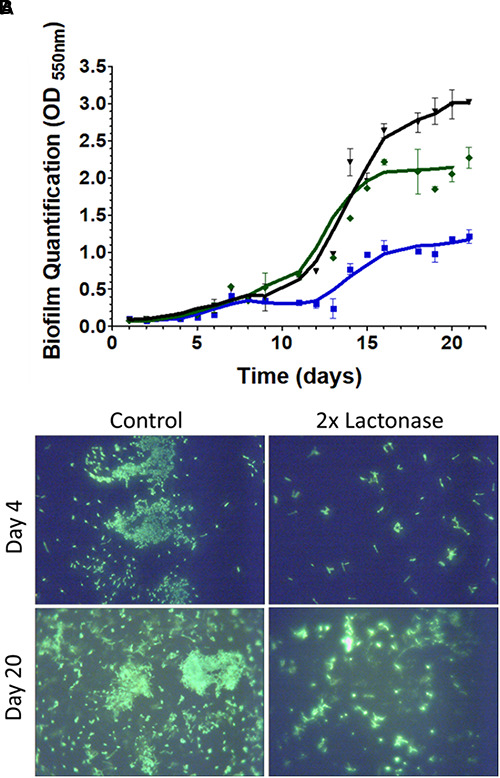
Presence of lactonase reduces biofilm formation of the microbial community in the bioreactors. **(A)** Bioreactor parameters over the time course of the experiment (21 days). Measurements (duplicates) were performed on the three distinct bioreactors equipped with different filtration cartridges: the 2× lactonase cartridge, containing only *lactonase beads* (blue line), the control cartridge containing only *control beads* (dark line), and the 1× lactonase cartridge containing a 1:1 ratio of *lactonase beads* and *control beads* (green line). Biofilm quantification in submerged wells as quantified by crystal violet binding measured at 550 nm. **(B)** Glass slips submerged in the bioreactors were stained using SYBR gold DNA stain and visualized using a 60× magnification.

Biofilm formed and was quantified over the time course of the experiment in the bioreactors (Figure 3A and Supplementary Figure S3). Submerged plastic wells were sampled and assayed using crystal violet dye (Supplementary Figure S4). Measurements indicated that biofilms were slowly forming during the first 11 days of the experiments, and then accelerated in all bioreactors. Interestingly, there were no significant differences in biofilm quantification during the first 11 days of the experiment between filtration systems using lactonase or control beads. However, after 23 days in the presence of the lactonase beads, biofilm was reduced to ∼50% of that formed in the control. Biofilm reduction might even be greater since OD_600__nm_ measurements reached saturation in the control experiment. The degree of biofilm reduction is consistent with the observed reduction in biofilm dry weight in tubing [49–44% when comparing control and 2× lactonases after 21 days for pre-column and post column tubing, respectively (Supplementary Figure S5)]. Remarkably, inhibition of biofilm was observed as a function of lactonase concentration in the cartridge: inhibition was larger in the 2× concentration compared to the 1× concentration (∼50% and ∼30%, respectively) (Figure 3A). The reasons accounting for this enzyme dose dependence are unclear at this stage, and will be the subject of subsequent investigations in greater details.

Biofilm formation in the bioreactors was imaged in both the early and late stages of the experiment [days 4 and 20, respectively (Figure 3B)]. DNA staining of the submerged microscope slips revealed that the presence of lactonase in the filtration cartridge led to a reduction in the adhesion of cells to the coverslip surfaces. While we were unable to visualize the matrix in this experiment, it was apparent that the biofilms in the control tanks had more structure and maturity than did those formed in the lactonase treated tank. Interestingly, the reduction of cell attachment was also observed in the early stage of biofilm formation (by day 4) and may indicate the importance of signaling in both the biofilm attachment and maturation steps (Parsek and Greenberg, 2005). The presence of bacteria in our tested community, such as *Pseudomonas* and *Aeromonas* that are known to form biofilms, to utilize AHL-based QS and to be inhibited by lactonases (Cao et al., 2012; Guendouze et al., 2017), may partly explain the observed biofilm inhibition.

The ability of lactonase to inhibit complex biofilms was first evidenced using encapsulated microbes naturally expressing lactonases in MBR systems (Kim et al., 2013). Here, our study provides a comprehensive analysis of the dynamics of the effect of lactonase on a soil community, and with two different doses of the enzymatic quencher. Additionally, the demonstration in this study of the ability of entrapped, overexpressed and improved lactonases to inhibit biofilm formation in a recirculating system opens new perspectives for enzyme technology use in water treatment. It also raises questions about the specific mechanism of action of entrapped lactonases on the microbial community signaling. Because lactonase enzymes degrade the secreted signaling molecules (AHLs), no physical contact between the enzyme molecules and bacteria might be needed for its action. This hypothesis is consistent with our bioreactor design and our observed inhibition of biofilm formation. Questions concerning the diffusion ability of AHLs in various media will need to be investigated, as it may modulate the “action range” of the various AHLs, and consequently, of lactonases.

### The Presence of a Lactonase Induces Changes in the Biofilm Microbial Composition

**FIGURE 4:**
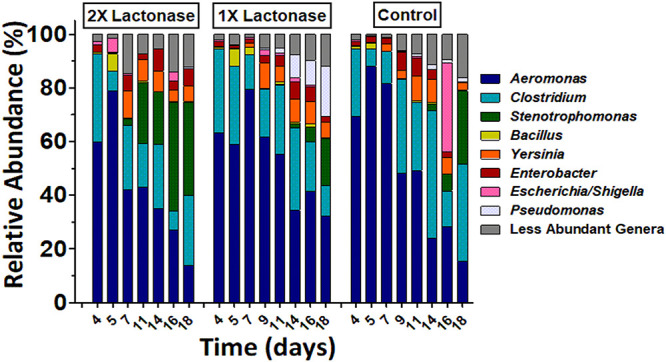
Biofilm bacterial community changes as a function of lactonase concentration and time. Relative abundance of bacteria at the genus level in the three different bioreactors (2× lactonase; 1× lactonase and control) over time (from day 4 to day 18).

**FIGURE 5:**
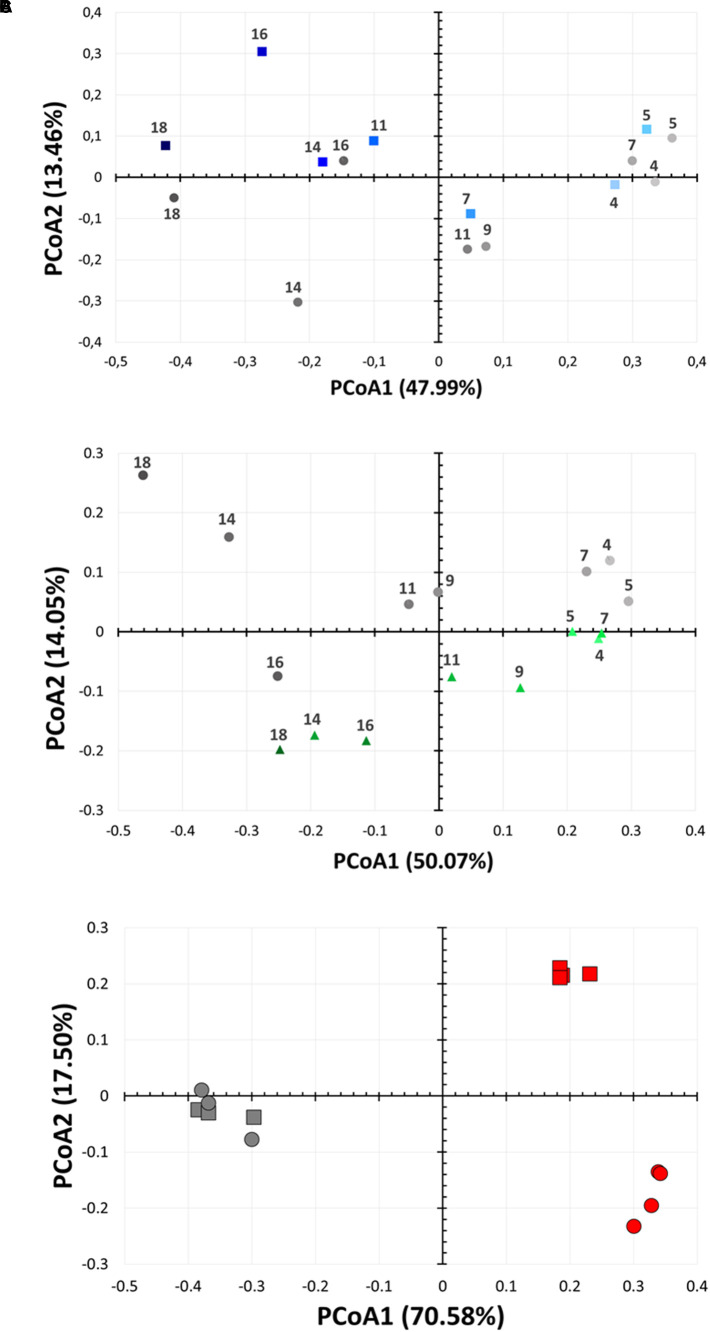
Bacterial community changes as a function of lactonase concentration and time. Principal coordinate analysis of microbial biofilm communities from the bioreactors over time (from day 4 to day 18). Analysis are performed for **(A)** the 2× lactonase (blue squares) and control communities (gray circles) and **(B)** the 1× lactonase (green triangles) and control communities (gray circles). **(C)** Principal coordinate analysis of suspension microbial communities from a small scale suspension culture in presence (red) or absence (gray) of active lactonase. Data (*n* = 4 replicates) for days 3 and 7 are shown as squares and circles, respectively. Control group (*n* = 3 replicates) was treated with the inactive mutant SsoPox 5A8.

DNA from biofilm samples obtained from the three different bioreactors were isolated and submitted for amplicon sequencing of 16S rRNA (Figure 4). Samples were collected over the time course of the experiment to evaluate the population dynamics. Given the samples had relatively low diversity, we analyzed 1,250 sequences from each sample. This low diversity may be due to the pre-culture step of soil samples that reduced microbial diversity, as described in other examples (Tabacchioni et al., 2000; Gorski, 2012). Taxonomic community composition, classified to genus, (Figure 4) and principle coordinate analysis (Figure 5 and Supplementary Figure S6) indicated that microbial communities in all three replicate systems were very similar in the early stages of the experiment (ANOSIM, *p* > 0.05). For example, at day 4, *Aeromonas* represents 59.76, 63.20 and 69.52% of 2×, 1×, and control treatments, respectively. This was expected because all three bioreactors were inoculated with the same starting culture. However, notable differences in biofilm microbial communities were seen from day 7. By day 7, 42.2% of the microbial communities in biofilms from bioreactors treated with the highest concentration of lactonase (2×) were comprised by *Aeromonas*. In contrast, this bacterium comprised 79.7% and 81.7% of the community in the lactonase (1×) and control treatments, respectively (Figure 4). Principle coordinate analysis also highlighted that community composition starts to separate from control by day 7 (ANOSIM, *R* = 0.625, *p* = 0.036) (Figure 5).

Other notable differences include the relative populations of *Stenotrophomonas* (Gram-negative)*, Pseudomonas* (Gram-negative), and *Clostridium* XIVa (Gram-positive) (Figure 4). For instance, the 2× lactonase bioreactor showed that *Stenotrophomonas* appeared much earlier than that seen in the 1× lactonase and control bioreactors (on day 14). In the 1× lactonase bioreactor, however, we observed an increase in the *Pseudomonas* population by day 11 and a gradual increase in its abundance within the community throughout the rest of the experiment. Lastly, in the later part of the experiment (days 9–18), the control bioreactor hosted a larger *Clostridium* population than did the two other bioreactors.

These experiments were repeated as an independent experiment performed in duplicates with a community from the same soil location but sampled at a different time of the year. We used the 1× lactonase concentration, and sampled two time points (day 3 and day 10) (Supplementary Figure S7). Interestingly, despite the different starting community composition, we can observe similar features. Notable differences are seen by day 10, including an increase of the *Stenotrophomonas* population in the presence of the enzyme (8% *versus* 1.5% in control). The *Pseudomonas*, specifically observed in the presence of lactonase as described above, are also favored in this independent run (15.7% *versus* 1.4%, *p* < 0.05). Lastly, the *Clostridium* group also varies (4.5% *versus* 14.2%; *p* < 0.05) and is less abundant in presence of lactonase than in the control bioreactor. These three groups are similarly affected by signal disruption using the lactonase in both independent experiments.

These data reveal that the presence of the lactonase enzyme of the filtration cassette lead to changes in the composition of the communities that occurred rapidly and were persistent throughout the experiment. Data show that these alterations of microbial communities occur in a lactonase dose-dependent fashion, and can be observed in biofilm communities.

### The Presence of a Lactonase Induces Changes in the Microbial Composition in Suspension Culture

Experiments performed with the same starting community in smaller volumes (5 mL) of suspension cultures and in presence of the inactive 5A8 mutant or the lactonase *Sso*Pox W263I reveal similar alteration of the composition of the microbial community. Whereas in this experimental setup, no biofilm formation would be detected within the time frame (7 days), the analysis of sequencing data of culture supernatant samples to the genus-level (Supplementary Figure S8), PCoA (Figure 5C) and statistics (Supplementary Tables S1, S2) reveal that these suspension microbial communities are significantly different between treatments (ANOSIM, *R* = 0.94, *p* < 0.001). In fact, the compositions of the microbial population in presence or in absence of quorum quencher are so different that comparisons between communities are difficult. Nevertheless, we observe again that *Pseudomonas* are more abundant in the presence of the lactonase than in the control experiments (24.9% versus 14%; *p* < 0.05). These experiments reveal that the effects of signal disruption using the lactonase *Sso*Pox W263I robustly alters the composition of the treated microbial community and that changes are not limited to biofilms, but also affect suspension communities.

### Presence of a Lactonase Modulates Diversity Within Genera but Not the Community Diversity

**FIGURE 6:**
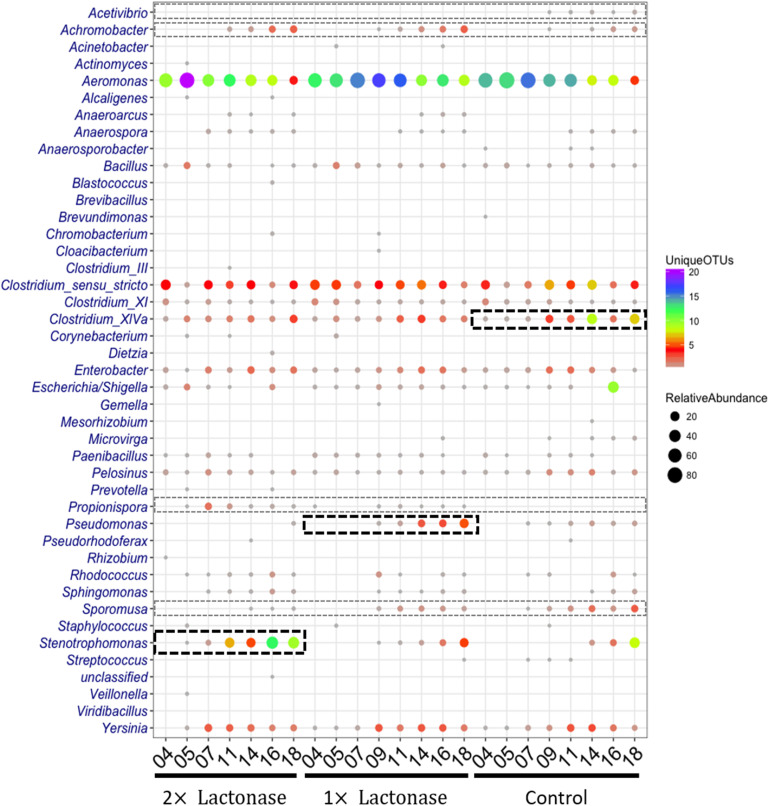
The shift of relative abundance and diversity of bacterial taxa as a function of lactonase concentration and time at the genus level. The color represents the number of unique OTUs and the size of the circles is propositional to the total number of sequences for each given taxonomic group and sample. Abundant and less abundant genera showing difference in the bioreactors are shown in bold black-hashed boxes and light gray hashed boxes, respectively.

Analysis of the relative abundance and diversity of genera distinctly highlighted population changes as a function of lactonase concentration and time of incubation (Figure 6). Overall, this analysis showed that while the presence of the lactonase induced changes in the relative abundance and diversity of some bacterial genera, it did not significantly alter overall community diversity. This is further evidenced by Shannon indices and observed species counts that are both slowly increasing over the time course of the experiments in all three bioreactors in a similar way (Supplementary Figure S9). Additionally, this analysis (Figure 6) highlights that *Stenotrophomonas*, *Pseudomonas* and *Clostridium XIVa* are specifically enriched over time in the 2× lactonase (Pearson correlation, *r* = 0.95, *P* = 0.001), the 1× lactonase (Pearson correlation, *r* = 0.90, *P* = 0.002), and the control bioreactors (Pearson correlation, *r* = 0.77, *P* = 0.027), respectively. This abundance increase is concomitant with an increase in their diversity.

Bacterial community compositions revealed that a few genera in low abundance were specific to treatments. For example, *Propionispora* were only detected in flow systems using lactonase in the filtration cartridge, whereas *Acetivibrio* were only detected in the control bioreactor. Other microbial community biases were also noted: *Achromobacter* was more abundant in tanks using lactonases, as compared to controls. In contrast, the abundance and diversity of *Sporomusa* decreased as the concentration of lactonase increased.

Changes in microbial communities in the presence of lactonase were recently observed in the context of membrane biofouling (Jo et al., 2016), as well as fish gut microbiomes (Zhou et al., 2016). In this study, we show the dynamics of community composition changes in a water recirculating system and observe that these changes are concomitant with biofilm inhibition. Furthermore, these changes depend on the enzyme concentration and are related to the abundance and diversity of genera, but not the overall diversity of the community. The mechanism(s) underpinning the ability of QQ lactonases to affect complex microbial communities are unknown. Complete QS circuits (a synthase and a receptor) were previously reported to be found only in Proteobacteria (Case et al., 2008). Within the bacterial genera detected in this study, some are known to: (1) produce AHLs and utilize them for sensing [i.e., *Pseudomonas, Aeromonas, Yersinia,* (Venturi, 2005; Medina-Martínez et al., 2007; Ortori et al., 2007; Khajanchi et al., 2011)], (2) be capable of producing AHLs [i.e., *Enterobacter* (Yin et al., 2012; Ochiai et al., 2013)], and (3) be capable of sensing AHLs [i.e., *Stenotrophomonas, Escherichia, Shigella* (Soares and Ahmer, 2011; Martínez et al., 2015; Taghadosi et al., 2015; Lu et al., 2017)] (Supplementary Table S3). Others, however, are known to not produce, use, or sense AHLs (i.e., *Clostridium*). Additionally, the relationship between the AHL signal disruption by a lactonase and some genera is not straight forward as indicated by the increase of *Pseudomonas* in the community present in the 1× lactonase system. Furthermore, it is intriguing to note that *Clostridium* XIVa, despite being a Gram-positive bacterium that does not produce and/or sense AHLs, is reduced in the presence of the lactonase. This observation might echo previous studies describing the ability of lactonase to inhibit the biofilm of *Staphylococcus aureus* and *Escherichia coli* (Ivanova et al., 2015a; Mayer et al., 2015). Mechanisms explaining these observations are lacking. Yet, this study is a first comprehensive, time-resolved, statistically significant description on the effects of a lactonase on microbial community structures, and will inform the understanding of these complex interactions.

## Conclusion

Our study demonstrates that lactonase-containing beads reduce biofilm formation by a complex soil microbial community, in a dose-dependent manner. Biofilm inhibition was observed, despite the presence of abundant microbes that are not known for using or sensing AHLs, such as *Clostridium*. Sequencing analysis revealed that biofilm inhibition was concomitant to a change in the microbial community composition on the surface. Dynamic population analysis shows that the bias introduced by AHL signal disruption occurs rapidly and is persistent over the time course of the experiment. We show in three independent experiments that signal disruption using a lactonase robustly and significantly changes the composition of microbial communities. We show that changes related to the relative proportion of some genera, but may also reflect in the observed specific presence or absence of genera in the biofilm. Therefore, signal disruption using a lactonase may have global effects on microbial populations, and not only inhibit bacteria utilizing AHLs for signaling.

Additionally, we find that signal disruption also lead to changes in the composition of suspension communities. This suggests that the importance of AHLs signaling extends beyond biofilm formation. In fact, this unexpected finding likely points to the importance of signaling in the competition between bacteria within communities (Diggle et al., 2007; Sandoz et al., 2007; Defoirdt et al., 2010).

Finally, the system used in this study provides a unique platform to study the importance of bacterial signaling, and the effects of signal disruption on complex microbial communities of multiple origins. We strongly feel that these findings and tools will pave the way for future investigations exploring the potential use of QQ enzymes in the water treatment arena, as well as the importance of signaling in complex microbial communities.

## Data Availability

The datasets generated for this study can be found in Sequence Read Archive, SRP156219.

## Author Contributions

ME conceived and designed the work. MS, CB, and JS performed the experiments. ME, MS, CB, CS, QZ, MJS, and AA analyzed the data. QZ, MJS, and CS performed the statistical analysis. ME, CB, and MS wrote the first draft. ME, MS, JS, QZ, and CS wrote sections of the manuscript. ME, MJS, and AA critically revised the manuscript. All authors read and approved the final manuscript.

## Conflict of Interest Statement

ME is the co-founder, Scientific Advisory Board member, and equity holder of Gene&Green TK, a company that holds the license to the patent WO2014167140 A1. These interests have been reviewed and managed by the University of Minnesota in accordance with its Conflict of Interest policies. The remaining authors declare that the research was conducted in the absence of any commercial or financial relationships that could be construed as a potential conflict of interest.
