# Corrigendum: Signal Disruption Leads to Changes in Bacterial Community Population

**DOI:** 10.3389/fmicb.2021.691552

**Published:** 2021-05-10

**Authors:** Michael Schwab, Celine Bergonzi, Jonathan Sakkos, Christopher Staley, Qian Zhang, Michael J. Sadowsky, Alptekin Aksan, Mikael Elias

**Affiliations:** ^1^Department of Biochemistry, Molecular Biology and Biophysics, University of Minnesota, Twin Cities, St. Paul, MN, United States; ^2^Biotechnology Institute, University of Minnesota, Twin Cities, St. Paul, MN, United States; ^3^Department of Mechanical Engineering, University of Minnesota, Twin Cities, St. Paul, MN, United States; ^4^Department of Surgery, University of Minnesota, Twin Cities, St. Paul, MN, United States; ^5^Department of Soil, Water, and Climate, University of Minnesota, Twin Cities, St. Paul, MN, United States; ^6^Department of Plant and Microbial Biology, University of Minnesota, Twin Cities, St. Paul, MN, United States

**Keywords:** quorum sensing, lactonase, biofilm, microbial community, silica encapsulation

In the original article, the conflict of interest statement was incorrectly filed. The Conflict of Interest Statement should have been as follows: “ME is the co-founder, Scientific Advisory Board member, and equity holder of Gene&Green TK, a company that holds the license to the patent WO2014167140 A1. These interests have been reviewed and managed by the University of Minnesota in accordance with its Conflict of Interest policies. The remaining authors declare that the research was conducted in the absence of any commercial or financial relationships that could be construed as a potential conflict of interest.”

The authors apologize for this error and state that this does not change the scientific conclusions of the article in any way.

