# Supplementary material for: Signal Disruption Leads to Changes in Bacterial Community Population
Source: Front Microbiol. 2019 Mar 29;10:611. doi: 10.3389/fmicb.2019.00611 (PMC6449428; doi:10.3389/fmicb.2019.00611)
Supplement: Supplementary file 1 [file Table_1.DOCX]

**Supplementary Material**

**Signal Disruption Leads to Changes in Bacterial Community Population.**

Michael Schwab^a,b^, Celine Bergonzi^a,b^, Johnathan Sakkos ^c^, Christopher Staley^b,d^, Qian Zhang ^b,e^, Michael J. Sadowsky^b,e,f^, Alptekin Aksan^b,c^ and Mikael Elias^a,b^ #

^a^ Department of Biochemistry, Molecular Biology & Biophysics

^b^ BioTechnology Institute

^c^ Department of Mechanical Engineering

^d^ Department of Surgery

^e^ Department of Soil, Water, and Climate

^f^ Department of Plant and Microbial Biology

University of Minnesota, Twin Cities campus, Minnesota, USA

# Correspondence email: mhelias@umn.edu

| Samples comparison | statistical factor | Among | Within | Total | p-value |
| --- | --- | --- | --- | --- | --- |
| Control_3-Control_7-SsoPox_3-SsoPox_7 | SS | 1.655 | 0.144 | 1.798 | <0.001 |
|  | df | 3 | 10 | 13 |  |
|  | MS | 0.552 | 0.014 |  |  |
|  | Fs | 38.393 |  |  |  |
| Control_3-Control_7 | SS | 0.032 | 0.093 | 0.125 | 0.279 |
|  | df | 1 | 4 | 5 |  |
|  | MS | 0.032 | 0.023 |  |  |
|  | Fs | 1.378 |  |  |  |
| Control_3-SsoPox_3 | SS | 0.624 | 0.041 | 0.665 | 0.035 |
|  | df | 1 | 5 | 6 |  |
|  | MS | 0.624 | 0.008 |  |  |
|  | Fs | 75.986 |  |  |  |
| Control_3-SsoPox_7 | SS | 0.826 | 0.084 | 0.91 | 0.025 |
|  | df | 1 | 5 | 6 |  |
|  | MS | 0.826 | 0.017 |  |  |
|  | Fs | 49.02 |  |  |  |
| Control_7-SsoPox_3 | SS | 0.618 | 0.059 | 0.677 | 0.033 |
|  | df | 1 | 5 | 6 |  |
|  | MS | 0.618 | 0.012 |  |  |
|  | Fs | 51.991 |  |  |  |
| Control_7-SsoPox_7 | SS | 0.828 | 0.103 | 0.931 | 0.028 |
|  | df | 1 | 5 | 6 |  |
|  | MS | 0.828 | 0.021 |  |  |
|  | Fs | 40.36 |  |  |  |
| SsoPox_3-SsoPox_7 | SS | 0.338 | 0.051 | 0.389 | 0.027 |
|  | df | 1 | 6 | 7 |  |
|  | MS | 0.338 | 0.008 |  |  |
|  | Fs | 39.779 |  |  |  |

SS = sum of square; df = degrees of freedom; MS = mean square; Fs = F Statistics

**Table S1**: AMOVA Statistical tests of suspension community sequencing data. Control 3 and 7 are for samples treated with the inactive mutant 5A8 and sampled at day 3 and 7, respectively. SsoPox 3 and 7 are for samples treated with the lactonase SsoPox W263I and sampled at day 3 and 7, respectively.

| Samples comparison | R-value | P-value |
| --- | --- | --- |
| Control_3-Control_7-SsoPox_3-SsoPox_7 | 0.94 | <0.001* |
| Control_3-Control_7 | 0.11 | 0.387 |
| Control_3-SsoPox_3 | 1.00 | 0.026 |
| Control_3-SsoPox_7 | 1.00 | 0.028 |
| Control_7-SsoPox_3 | 1.00 | 0.025 |
| Control_7-SsoPox_7 | 1.00 | 0.029 |
| SsoPox_3-SsoPox_7 | 1.00 | 0.024 |

**Table S2**: ANOSIM Statistical tests of suspension community sequencing data. Control 3 and 7 are for samples treated with the inactive mutant 5A8 and sampled at day 3 and 7, respectively. SsoPox 3 and 7 are for samples treated with the lactonase SsoPox W263I and sampled at day 3 and 7, respectively.

**Table S3**: Production and sensing of AHLs in representative strains from the main genus identified in the biofilm community.

| Detected Genera | Gram stain | Example strain | AHLs production | AHLs receptor | Reference |
| --- | --- | --- | --- | --- | --- |
| *Acetivibrio* | negative | N/A | N/A | N/A | N/A |
| *Achromobacter* | negative | *Achromobacter piechaudii* | N/A | Yes, and encodes a lactonase | (1, 2) |
| *Aeromonas* | negative | *Aeromonas hydrophilae* | C4 AHL; C6AHL | yes | (3) |
| *Clostridium* | Positive | N/A | N/A | N/A | N/A |
| Stenotrophomonas | negative | Stenotrophomonas maltophilia | N/A | yes | (4) |
| *Bacillus* | positive | *Bacillus subtilis BS-1* | N/A | No receptor, but encodes lactonases | (5) |
| *Yersinia* | negative | *Yersinia pseudotuberculosis* | 25 different AHLs, most abundant are 3-oxo C6, C7 and C8 AHLs | yes | (6, 7) |
| *Enterobacter* | negative | *Enterobacter sp. ; Enterobacter ludwigii*  *EN-119* | C12 AHL | N/A | (8, 9) |
| *Escherichia / Shigella* | negative | *Escherichia coli* | No production | yes | (10–12) |
| *Propionispora* | negative | N/A | N/A | N/A | N/A |
| *Pseudomonas* | negative | *Pseudomonas aeruginosa* | C4 AHL; 3-oxo C12 AHL | yes | (13) |
| *Sporomusa* | negative | N/A | N/A | N/A | N/A |

N/A (not applicable): data not available for this genera

**
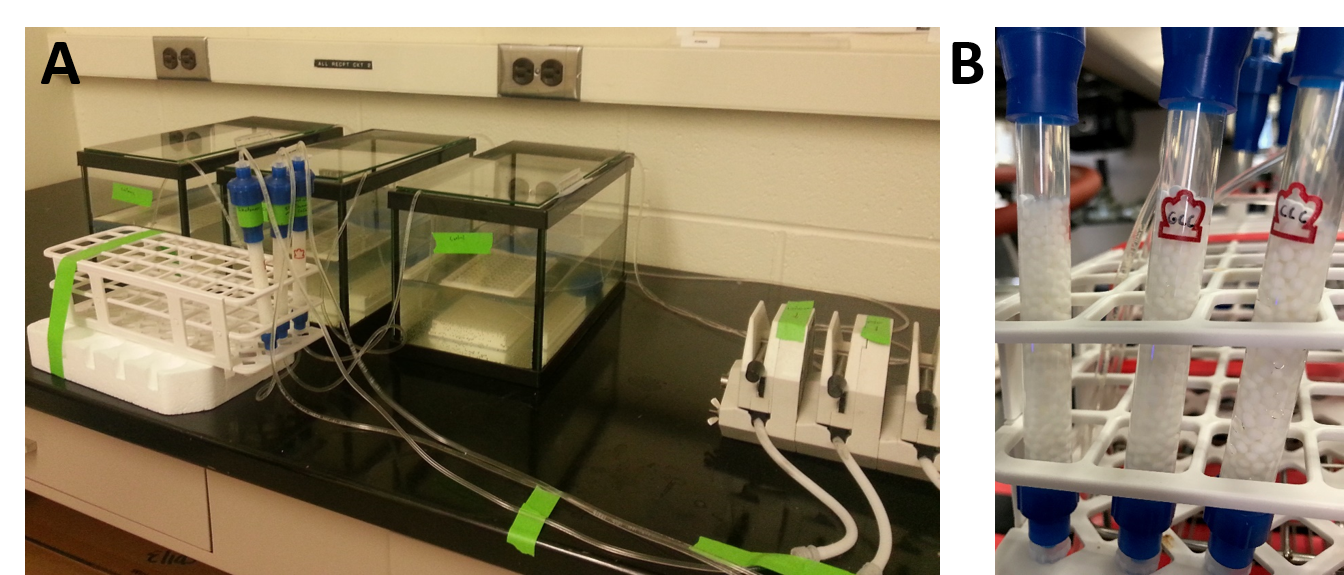
**

**Figure S1. Experimental bioreactor and filtration system. (A)** Bacterial communities are cultured in a tank vessel. A peristaltic pump is pumping the culture media through a filtration cartridge made of silica beads. (**B**) The beads are entrapping *E. coli* cells that overproduced an engineered quorum quenching lactonase, and used to fill up a column to serve as a filtration cartridge.

**
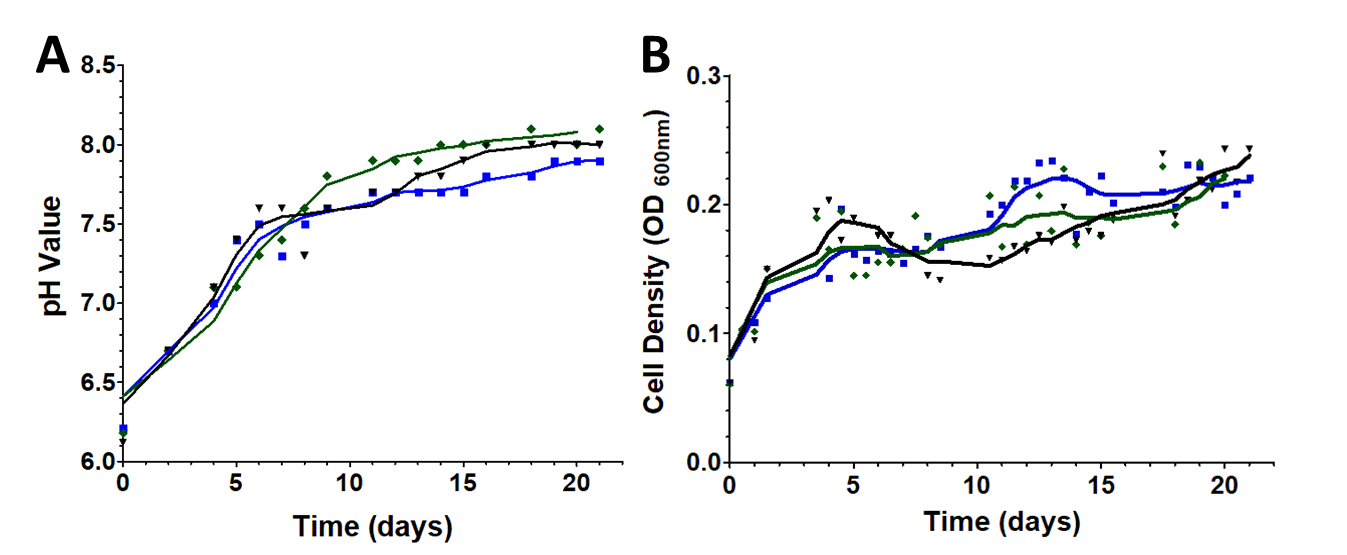
**

**Figure S2. Bioreactors’ pH values over the time course of the experiment (21 days).** Measurements were performed on the three distinct bioreactors equipped with different filtration cartridges: the 2× lactonase cartridge, containing only *lactonase beads* (blue line), the control cartridge containing only *control beads* (dark line) and the 1× lactonase cartridge containing a 1:1 ratio of *lactonase beads* and *control beads* (green line)*.* (**A**) pH monitoring over the time-course of the experiment. (**B**) Bacterial growth as measurement by the optical density at 600nm.


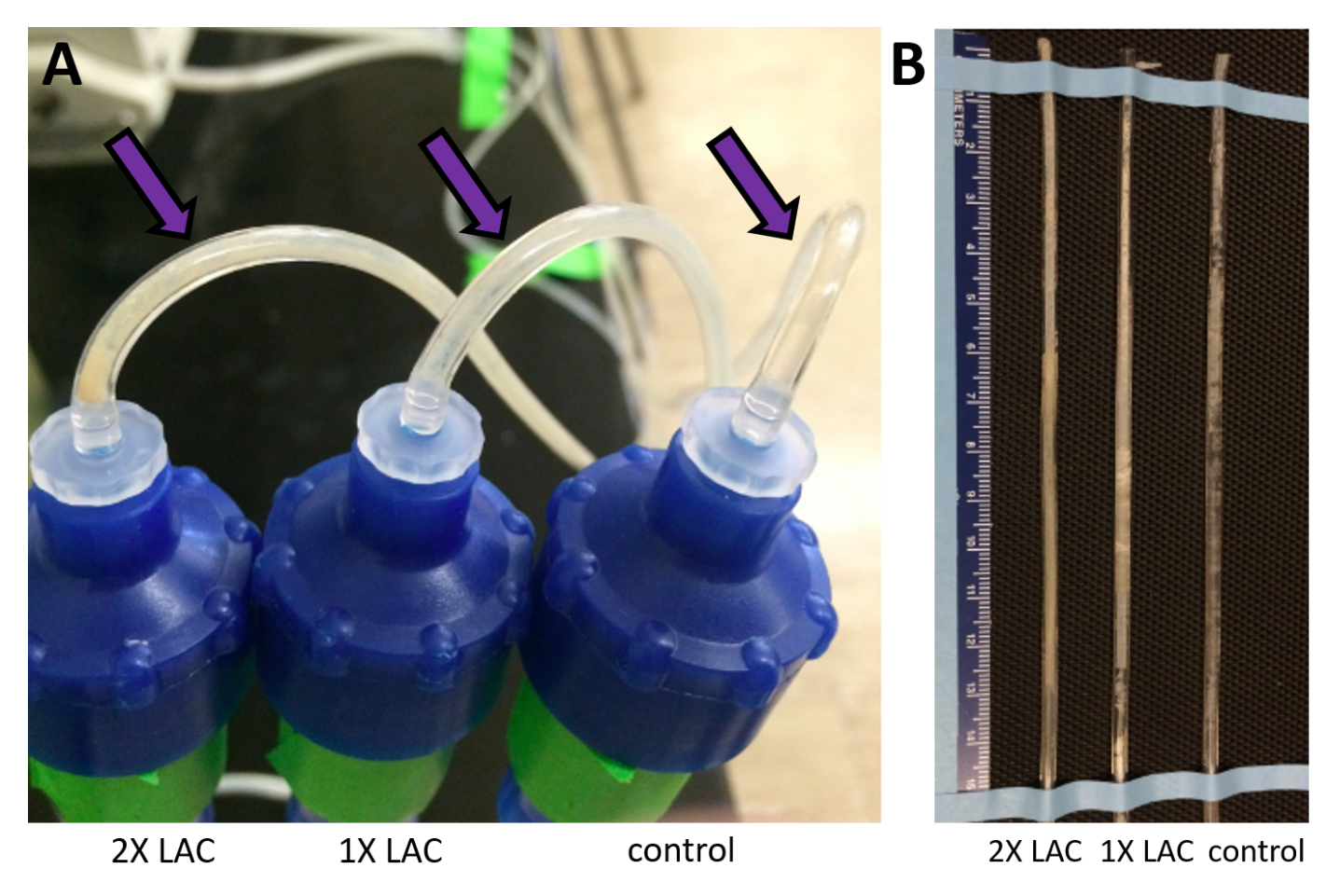


**Figure S3. Biofilm formation in tubing. (A)** Pre-column visible biofilm (purple arrow) for the different bioreactors (2×, 1× lactonase, control) after 14 days. (**B**) Tubing with dried biofilm at the end of the experiment.


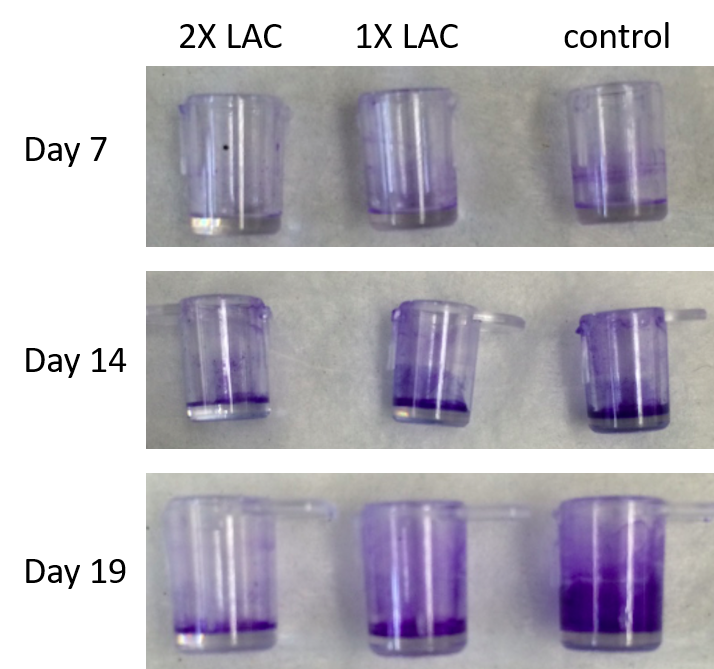


**Figure S4. Biofilm quantification using crystal violet dye.** 96-well plates with detachable wells were submerged in the bioreactors and wells were sampled at various times for biofilm quantification using crystal violet dye. Staining is visibly reduced in presence of the highest lactonase concentration (2× lactonase), as compared to the lower lactonase concentration (1×) and control.


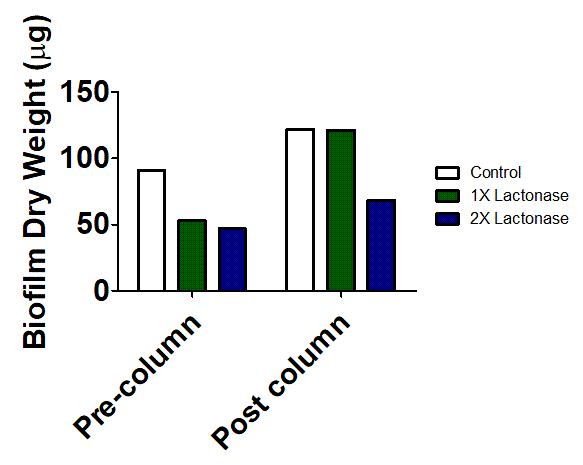


**Figure S5. Biofilm dry weight in tubing at the end of the experiment (21 days).** Sections of silicone tubing (15.0cm) were cut and dried for 24hours, and weighted on a precision balance. Dry weight are shown after subtracting the weight of a biofilm free section of identical tubing.


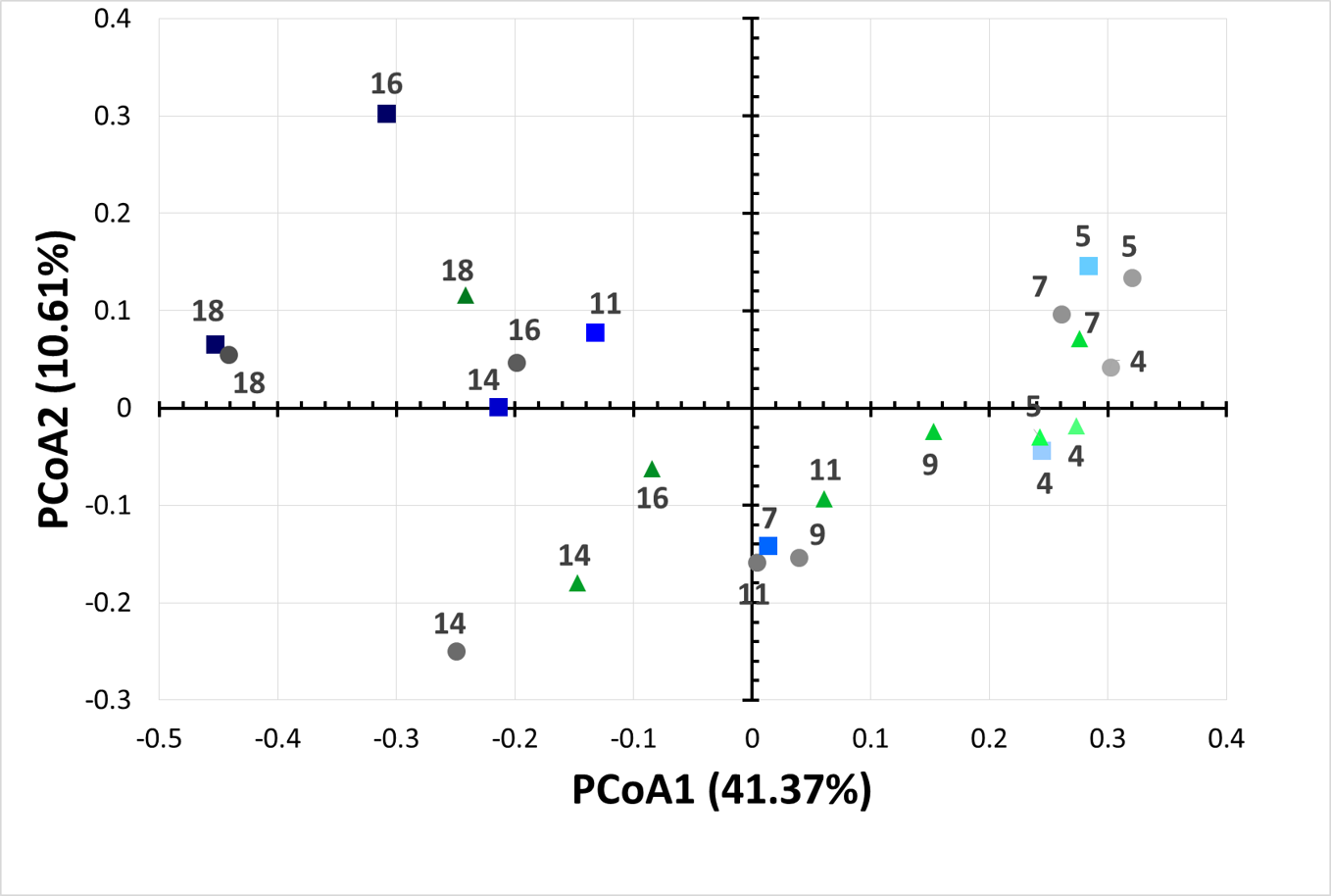


**Figure S6. Bacterial community changes as a function of lactonase concentration and time.** Principal coordinate analysis of microbial communities over time (from day 4 to day 18). Analysis are performed for the 2× (blue squares), 1× lactonase (green triangles) and control communities (grey circles).


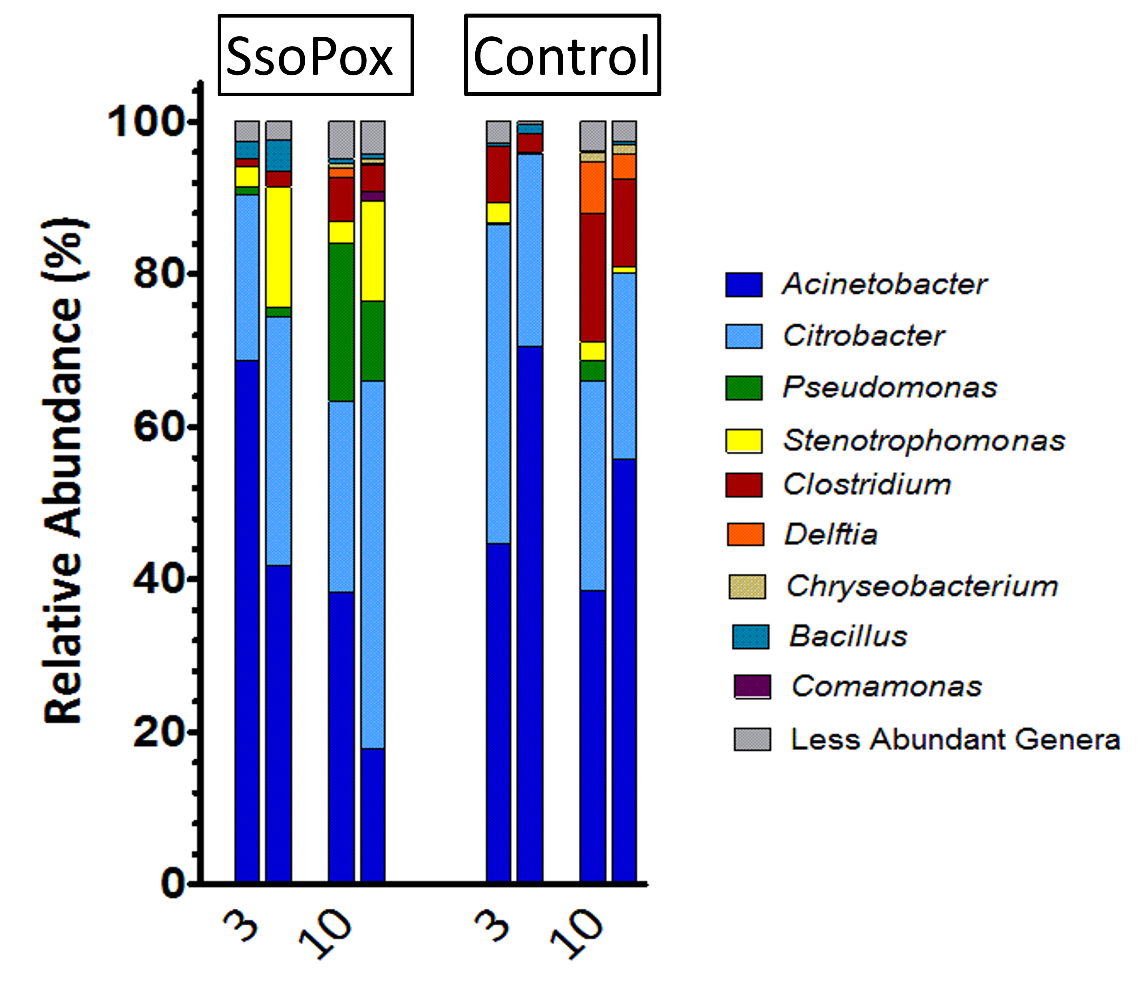


**Figure S7. Biofilm bacterial community changes as a function of time.** This is a second independent experiment in bioreactors relative to experiment in Figure 4. Relative abundance of bacteria at the genus level for community treated with the inactive enzyme 5A8 (control) and the active enzyme (SsoPox-W236I) at two different times (days 3 and 7).


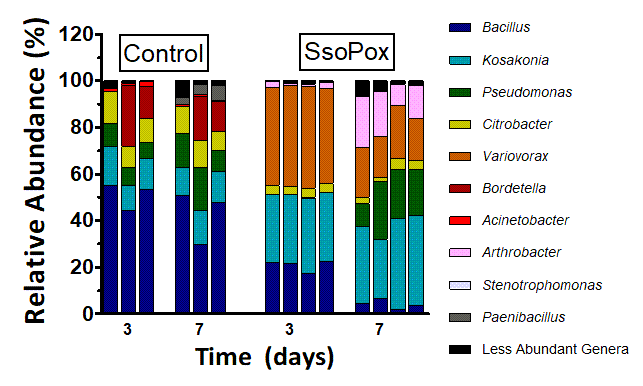


**Figure S8. Suspension bacterial community changes in presence or absence of active lactonase.** Analysis were performed using 16S v4 rRNA sequencing data. We note that despite the use of the same starting soil community, different growth setups yielded different community compositions than the bioreactor setup. Relative abundance of bacteria at the genus level for community treated with the inactive enzyme (control) and the active enzyme (SsoPox-W236I) at two different times (days 3 and 7).


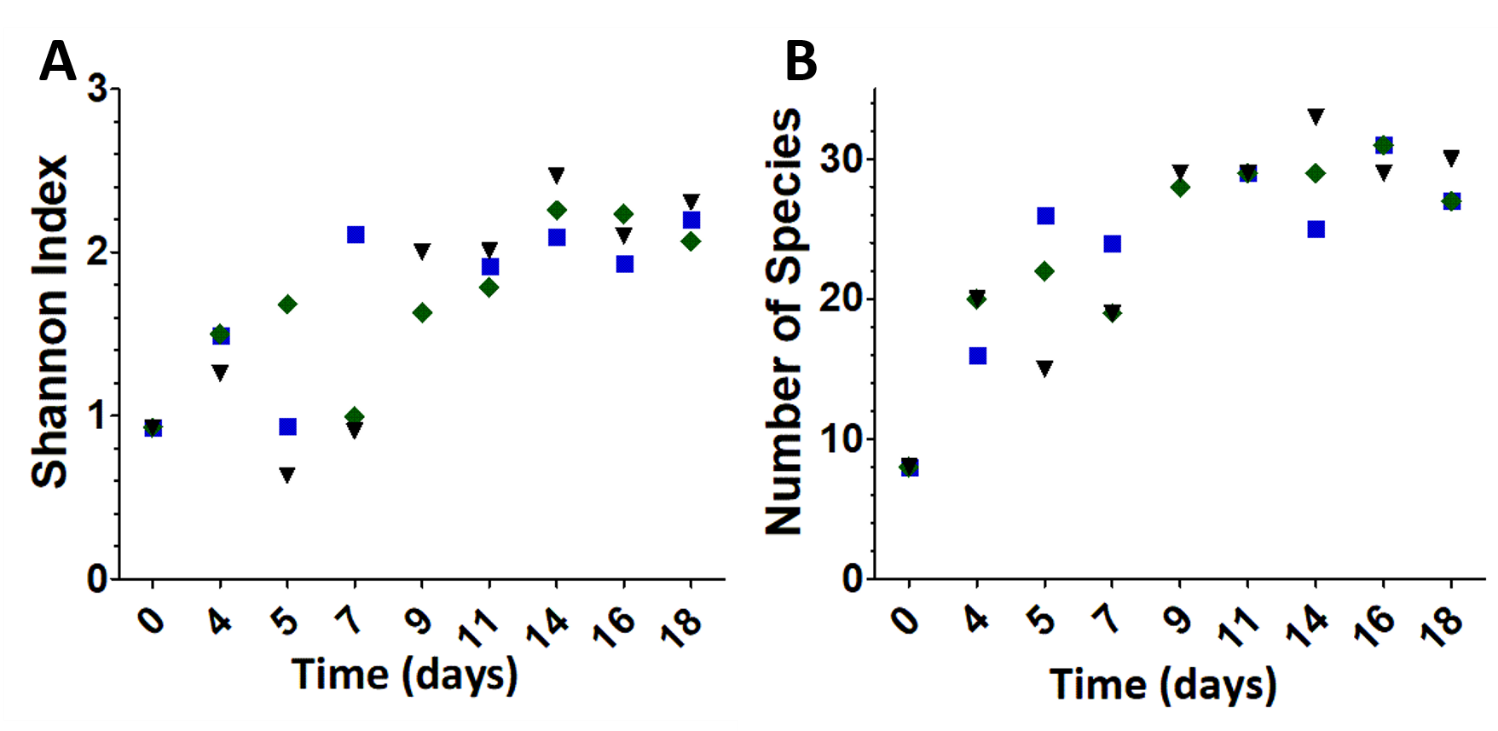


**Figure S9. Shannon index and number of observed species in the biofilm communities.** Blue squares, green diamonds and black triangles are for 2× lactonase, 1× lactonase and control treated bioreactors, respectively. (**A**) Shannon indexes were calculated for the different microbial communities over time (from day 4 to day 18). (**B**) Number of different species observed in the different microbial communities over time (from day 4 to day 18).

References

1. Kretzschmar AL, Manefield M. 2015. The role of lipids in activated sludge floc formation. AIMS Env Sci 2:122–133.

2. Swearingen MC, Sabag-Daigle A, Ahmer BM. 2013. Are there acyl-homoserine lactones within mammalian intestines? J Bacteriol 195:173–179.

3. Khajanchi BK, Kirtley ML, Brackman SM, Chopra AK. 2011. Immunomodulatory and protective roles of quorum-sensing signaling molecules N-acyl homoserine lactones during infection of mice with Aeromonas hydrophila. Infect Immun 79:2646–2657.

4. Martínez P, Huedo P, Martinez-Servat S, Planell R, Ferrer-Navarro M, Daura X, Yero D, Gibert I. 2015. Stenotrophomonas maltophilia responds to exogenous AHL signals through the LuxR solo SmoR (Smlt1839). Front Cell Infect Microbiol 5:41.

5. Pan J, Huang T, Yao F, Huang Z, Powell CA, Qiu S, Guan X. 2008. Expression and characterization of aiiA gene from Bacillus subtilis BS-1. Microbiol Res 163:711–716.

6. Ortori CA, Atkinson S, Chhabra SR, Cámara M, Williams P, Barrett DA. 2007. Comprehensive profiling of N-acylhomoserine lactones produced by Yersinia pseudotuberculosis using liquid chromatography coupled to hybrid quadrupole–linear ion trap mass spectrometry. Anal Bioanal Chem 387:497–511.

7. Medina‐Martínez M, Uyttendaele M, Meireman S, Debevere J. 2007. Relevance of N‐acyl‐L‐homoserine lactone production by Yersinia enterocolitica in fresh foods. J Appl Microbiol 102:1150–1158.

8. Ochiai S, Morohoshi T, Kurabeishi A, Shinozaki M, Fujita H, SAWADA I, Ikeda T. 2013. Production and degradation of N-acylhomoserine lactone quorum sensing signal molecules in bacteria isolated from activated sludge. Biosci Biotechnol Biochem 77:2436–2440.

9. Yin W-F, Purmal K, Chin S, Chan X-Y, Chan K-G. 2012. Long chain N-acyl homoserine lactone production by Enterobacter sp. isolated from human tongue surfaces. Sensors 12:14307–14314.

10. Lu Y, Zeng J, Wu B, Wang L, Cai R, Zhang N, Li Y, Huang X, Huang B, Chen C. 2017. Quorum sensing N-acyl homoserine lactones-SdiA suppresses Escherichia coli-Pseudomonas aeruginosa conjugation through inhibiting traI expression. Front Cell Infect Microbiol 7:7.

11. Taghadosi R, Shakibaie MR, Masoumi S. 2015. Biochemical detection of N-Acyl homoserine lactone from biofilm-forming uropathogenic Escherichia coli isolated from urinary tract infection samples. Rep Biochem Mol Biol 3:56.

12. Soares JA, Ahmer BM. 2011. Detection of acyl-homoserine lactones by Escherichia and Salmonella. Curr Opin Microbiol 14:188–193.

13. Venturi V. 2005. Regulation of quorum sensing in Pseudomonas. FEMS Microbiol Rev 30:274–291.
